# Emerging Chemical Biology of Protein Persulfidation

**DOI:** 10.1089/ars.2023.0352

**Published:** 2023-07-17

**Authors:** Thibaut Vignane, Milos R. Filipovic

**Affiliations:** Leibniz Institute for Analytical Sciences, ISAS e.V., Dortmund, Germany.

**Keywords:** protein persulfidation, hydrogen sulfide, redox signaling, aging, posttranslational modifications, cysteine

## Abstract

**Significance:**

Protein persulfidation (the formation of RSSH), an evolutionarily conserved oxidative posttranslational modification in which thiol groups in cysteine residues are converted into persulfides, has emerged as one of the main mechanisms through which hydrogen sulfide (H_2_S) conveys its signaling.

**Recent Advances:**

New methodological advances in persulfide labeling started unraveling the chemical biology of this modification and its role in (patho)physiology. Some of the key metabolic enzymes are regulated by persulfidation. RSSH levels are important for the cellular defense against oxidative injury, and they decrease with aging, leaving proteins vulnerable to oxidative damage. Persulfidation is dysregulated in many diseases.

**Critical Issues:**

A relatively new field of signaling by protein persulfidation still has many unanswered questions: the mechanism(s) of persulfide formation and transpersulfidation and the identification of “protein persulfidases,” the improvement of methods to monitor RSSH changes and identify protein targets, and understanding the mechanisms through which this modification controls important (patho)physiological functions.

**Future Directions:**

Deep mechanistic studies using more selective and sensitive RSSH labeling techniques will provide high-resolution structural, functional, quantitative, and spatiotemporal information on RSSH dynamics and help with better understanding how H_2_S-derived protein persulfidation affects protein structure and function in health and disease. This knowledge could pave the way for targeted drug design for a wide variety of pathologies. *Antioxid. Redox Signal.* 39, 19–39.

## Introduction: From Hydrogen Sulfide to Protein Persulfide

Ever since Abe and Kimura (1996) demonstrated that hydrogen sulfide (H_2_S) could act as a gasotransmitter (endogenously produced gaseous molecule with signaling properties), interest in H_2_S biology has been growing exponentially. However, H_2_S is also suggested to have played a crucial role in the origin of life itself. This constitutes the basis for the cyanosulfidic hypothesis of origin of life, where all building blocks of life (ribonucleotides, amino acids, and lipids) are created from cyanide and H_2_S with the help of ultraviolet light and traces of metal ions (Patel et al., 2015; Sasselov et al., 2020). The formation of cysteine from sulfide and cysteine's role as both a catalyst and precursor in prebiotic peptide synthesis have also been recently demonstrated, further emphasizing the importance of sulfur-based chemistry (Foden et al., 2020).

Early life-forms flourished in an H_2_S-rich environment. H_2_S was used for synthetic purposes, but also as a source of metabolic energy (Wang, 2012).

**FIG. 1.**
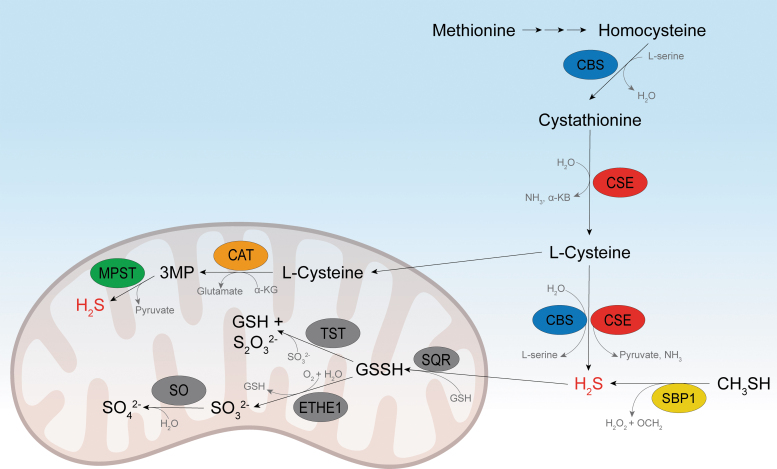
**Biosynthesis and oxidation of H_2_S.** α-KB, α-ketobutyrate; α-KG, α-ketoglutarate; 3-MP, 3-mercaptopyruvate; CAT, cysteine aminotransferase; CBS, cystathionine β-synthase; CSE, cystathionine *γ*-lyase; ETHE1, persulfide dioxygenase; GSH, glutathione; H_2_S, hydrogen sulfide; MPST, mercaptopyruvate sulfur transferase; SBP1 (alternatively SELENBP1), methanethiol oxidase; SO, sulfite oxidase; SQR, sulfide:quinone oxidoreductase; TST, thiosulfate sulfur transferase (alternatively rhodanese).

Some of those basic chemical principles played a role in the evolution of living systems and their successful maintenance, and so, it comes as no surprise that cells developed enzymes to control H_2_S production and consumption (Fig. 1). In the animal kingdom, H_2_S is produced in the transsulfuration pathway by the action of the pyridoxal 5′-phosphate (PLP)-dependent enzymes cystathionine β-synthase (CBS) and cystathionine γ-lyase (CSE; also known as CTH), but it can also be produced in the cysteine catabolism pathway by cysteine aminotransferase/mercaptopyruvate sulfurtransferase (MPST) (Fig. 1) (Filipovic et al., 2018; Kabil and Banerjee, 2014; Kabil et al., 2011). In addition, a fourth enzyme has been described, methanethiol oxidase (SELENBP1); it converts methanethiol into formaldehyde, hydrogen peroxide (H_2_O_2_), and H_2_S (Pol et al., 2018). Differentially expressed in different tissues (and even cellular compartments), these enzymes control H_2_S production with different efficiencies.

It has been estimated that the flux of sulfur into H_2_S is similar to that directed into the flux of glutathione (GSH) production (Kabil et al., 2011; Vitvitsky et al., 2012). However, the intracellular steady-state levels of H_2_S are quite low (submicromolar for most tissues) (Filipovic et al., 2018), suggesting very high clearance and/or consumption. H_2_S is primarily oxidized in the mitochondria by sulfide:quinone oxidoreductase (SQR), yielding glutathione persulfide (GSSH), which can be further processed either by thiosulfate sulfurtransferase (TST, also known as rhodanese) or by persulfide dioxygenase (ETHE1) (Fig. 1) (Filipovic et al., 2018; Kabil and Banerjee, 2014; Kabil et al., 2011). How all these enzymes control the spatiotemporal production/distribution of H_2_S is still unclear, but knockout of some of them has been shown to have a clear phenotype in experimental animals.

CSE knockout mice show abnormal hind limb clasping and clenching reminiscent of Huntington's disease (Paul et al., 2014). Mice deficient in CBS suffer from severe growth retardation, and a majority of them die by 5 weeks of age. TST and MPST knockout mice develop obesity (Katsouda et al., 2022; Morton et al., 2016), while the knockout of ETHE1 in mice and the loss of its expression in humans result in ethylmalonic encephalopathy (Tiranti et al., 2009), suggesting that tight regulation of H_2_S production and consumption is important for normal cellular function.

In the last two decades, H_2_S has been shown to play important roles in many biological processes, including blood pressure regulation (Eberhardt et al., 2014; Szijártó et al., 2018; Yang et al., 2008), angiogenesis (Das et al., 2018; Papapetropoulos et al., 2009; Szabó and Papapetropoulos, 2011), hypoxia sensing (Peng et al., 2010; Yuan et al., 2015), inflammation (Li et al., 2005; Whiteman and Winyard, 2011), neurodegeneration (Giovinazzo et al., 2021; Paul et al., 2014; Vandiver et al., 2013), and aging (Hine et al., 2015; Zivanovic et al., 2019). Biological chemistry, (patho)physiological roles, and pharmacological potentials of H_2_S have been the topic of many excellent review articles (Benchoam et al., 2019; Filipovic et al., 2018; Nagy et al., 2014; Ono et al., 2014; Szabó, 2007; Szabó and Papapetropoulos, 2011; Wallace and Wang, 2015; Wang et al., 2015) and are not discussed here in detail.

One of the main mechanisms *via* which H_2_S is believed to convey its biological effects is protein persulfidation, an oxidative posttranslational modification in which thiol groups (RSH) in cysteine residues are converted into persulfides (RSSH), and in this review, we focus on current advances in studying protein persulfidation, their biological roles, and on some open questions that this research field is facing.

## Biological Chemistry of Protein Persulfides

Protein persulfides are important intermediates in metabolic pathways requiring sulfur transfer, such as those leading to the formation of iron–sulfur clusters, biotin, thiamine, lipoic acid, molybdopterin, and sulfur-containing bases in RNA (Mueller, 2006). The seminal work from Snyder's group introduced protein persulfidation as a more widespread, general modification (Mustafa et al., 2009), providing the context for understanding the physiological and pharmacological effects of H_2_S (Filipovic et al., 2018; Paul and Snyder, 2015; Paul and Snyder, 2012).

The initial term that was introduced to name this modification was “sulfhydration” (Mustafa et al., 2009); however, this term was ambiguous since no water molecule is introduced as the word “hydration” would imply. The process instead involves “sulfuration” because it involves the addition of a sulfur atom. The name “persulfidation” has gained the widest acceptance, although “hydropersulfide” is also in use. The IUPAC has suggested the names hydridodisulfide, disulfane, or dithiohydroperoxide. In this article, “persulfide” is used for the mixture of RSSH and RSS^−^ groups in aqueous solution at a certain pH, unless otherwise specified.

In terms of their chemical reactivity, persulfides have characteristics in common with thiols, disulfides, polysulfides, hydroperoxides, and sulfenic acids. This is one of the main reasons why dissecting their specific biological functions is difficult.

### Acidity and nucleophilicity

Hydropersulfides, the protonated form of persulfides, can ionize to form the corresponding anionic persulfides [Eq. (1)].


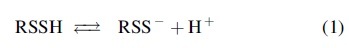


The S-H bond found in persulfides is weaker when compared with the corresponding thiol (Filipovic et al., 2018); therefore, the acidity of persulfides is expected to be higher. A computational estimate of the difference between the p*K*_a_ of cysteine persulfide and cysteine gave a value of −4, which suggests that the p*K*_a_ of cysteine persulfide is 4.3 (Cuevasanta et al., 2015). Recently, Alvarez's group experimentally determined the p*K*_a_ of GSSH to be 5.45, 3.49 units below that of GSH (Benchoam et al., 2020). All these data suggest that in the range of physiological pH values, persulfides exist almost completely as anionic species. It is worth noting that in the case of proteins, interaction with other functional groups surrounding persulfidated cysteine residue likely modulates its p*K*_a_.

Although the basicity represents only a particular affinity toward H^+^, the nucleophilicity and basicity are often correlated, since the stronger the basicity, the higher the nucleophilicity. Following that rule, RSSH should be weaker nucleophiles than RSH. However, a notable exception to this rule occurs when a vicinal (adjacent) atom carries a nonbonding electron pair. This has classically been referred to as the alpha effect (*e.g.*, HOO^−^ is more nucleophilic than HO^−^). Thus, the presence of the alpha effect in RSSH makes them more nucleophilic than RSH. In addition, the lower p*K*_a_ keeps RSSH in the correct ionized state for the reaction with electrophiles [Eq. (2)].

(2) $$RSS^{-}+E^{+}\to RSSE$$

An illustration of this “super nucleophilic” feature of RSSH is the apparent rate constant for the reaction of albumin persulfide with an electrophile, 4,4′-dithiodipyridine, which is ∼20 times higher than that of albumin's thiol (Cuevasanta et al., 2015). Persulfide of the one-cysteine peroxiredoxin (Prx) alkyl hydroperoxide reductase E from *Mycobacterium tuberculosis* reacted 43 times faster than did the RSH form of this protein (Cuevasanta et al., 2019). The nucleophilicity of RSSH is best demonstrated by their reaction with thiol alkylating agents, different disulfides, and electrophiles, such as 8-nitroguanosine 3′,5′-cyclic monophosphate (Artaud and Galardon, 2014; Ida et al., 2014) and methyl mercury (Abiko et al., 2015). Although RSH can also react with most of those substrates, RSSH do so faster.

### Electrophilicity and stability

**FIG. 2.**
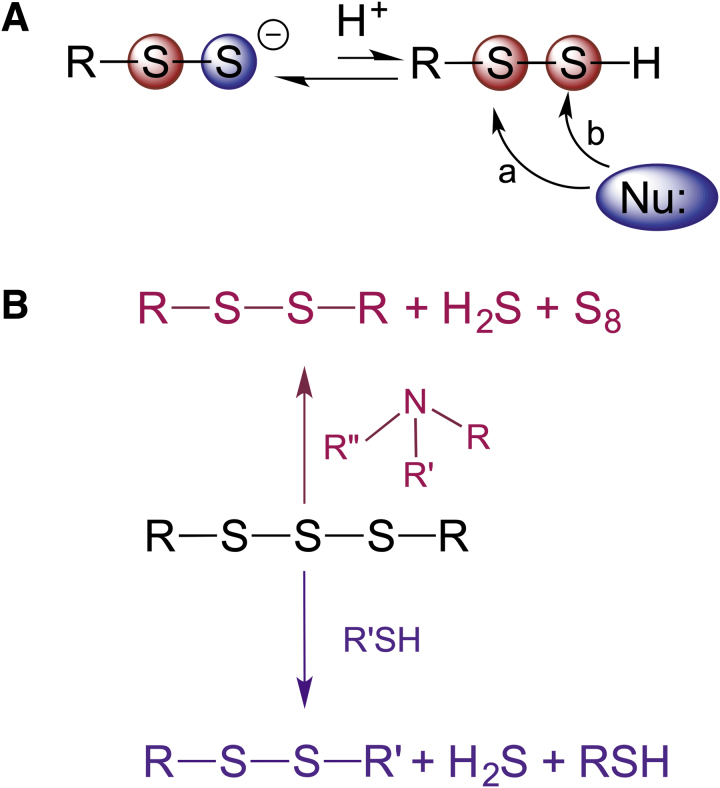
**Electrophilic nature of organic persulfides and polysulfides. (A)** Nucleophilic attack on electrophilic sulfur (*red circle*) of RSSH could occur on either of the two sulfur atoms with reaction path *a* being favored most of the time. **(B)** Instability of organic trisulfides is illustrated by their amine-induced or thiol-induced decomposition.

RSSH are sulfane sulfur-containing compounds. Sulfane sulfur is defined as a sulfur atom bound to two sulfurs or to a sulfur and an ionizable hydrogen (Filipovic et al., 2018). Therefore, in their protonated form, RSSH are weak electrophiles. When reacting with a nucleophile, nucleophilic attack can occur either on the inner sulfur, releasing H_2_S, or on the outer sulfur with elimination of the thiol (Fig. 2A). Unless the protein environment surrounding cysteine residues influences the electron density/electrophilicity of two sulfur atoms, the former (Path a, Fig. 2A) is always preferred over the latter (Path b, Fig. 2A). The reason for this is that H_2_S has a lower p*K*_a_ (6.98) than the average p*K*_a_ of thiol (p*K*_a_ 8–9), which makes it a much better leaving group.

A notable exception to this rule would be the reaction of an RSSH with cyanide (Wood, 1987), where thiocyanate is formed [Eq. (3)]. During evolution, this reaction was used for enzymatic cyanide detoxification by rhodanese (TST).

(3) $$RSSH+CN^{-}\to RSH+SCN^{-}$$

In regard to the reaction with thiols, experiments with either low-molecular-weight (LMW) persulfides (Artaud and Galardon, 2014; Bailey et al., 2014; Kawamura et al., 1966) or protein persulfides (Pan and Carroll, 2013) resulted in the release of H_2_S. This is important to highlight because thiols are present at millimolar concentrations inside cells. Recent work from Murphy's group on the development of the LMW mitochondria-targeted RSSH MitoPerSulf demonstrates how this molecule, when used in a biological context, readily reacts with GSH to release H_2_S (Miljkovic et al., 2022). However, some protein persulfides formed in the active site of enzymes can react with thiols following Equation (4). This reaction is called transpersulfidation (*vide infra*).

(4) $$RSSH+R\prime S^{-}\to RSH+R\prime SS^{-}$$

Both the nucleophilic nature and electrophilic nature of RSSH make them very unstable in solution and difficult to work with. For example, the real-time mass spectrometry (MS) of *N*-acetyl penicillamine persulfide showed that it decays with a half-life of 2.7 min (Wedmann et al., 2016); slightly higher values have been reported for the decay of CysSSH (Yadav et al., 2016). In both cases, the decay predominantly occurred through the reaction shown in Equation (5).

(5) $$RSSH+RSS^{-}\to RSSSR+RS^{-}$$

Of note, the trisulfides formed in this reaction are gaining much attention as potential precursors of RSSH (Barayeu et al., 2022; Bianco et al., 2019). However, cysteine and glutathione trisulfide are relatively unstable under physiological conditions, undergo amine-induced decomposition (Fig. 2B) (Brown and Bowden, 2022), and act as electrophiles, directly modifying cysteine residues (Switzer et al., 2021), and so, their biological effects should not be equated to those of RSSH.

### Reaction with one- and two-electron oxidants: chemical basis for antiferroptotic and antioxidant effects

RSSH have a lower energy of dissociation of the S-H bond than thiols (293 kJ/mol *vs.* 385 kJ/mol) (Filipovic et al., 2018) and a much more favorable one-electron reduction potential [*E*°′(RSS^•^/RSS^−^) = 0.68 V *vs. E*°′(RS^•^, H^+^/RSH) = 0.96 V] (Koppenol and Bounds, 2017), which makes them good one-electron reductants. Furthermore, in perthiyl radicals, the unpaired electron is delocalized between the two sulfur atoms, leading to resonance stabilization and increased stability of these radicals (Everett and Wardman, 1995; Everett et al., 1994). Depending on the one-electron oxidant, they can be involved in either electron transfer or hydrogen atom transfer [Eqs. (6) and (7)].

(6) $$RSS^{-}+{A_{2}}^{∙}\to RSS^{∙}+{A_{2}}^{-}$$

(7) $$RSSH+{A_{1}}^{∙}\to RSS^{∙}+A_{1}H$$

**FIG. 3.**
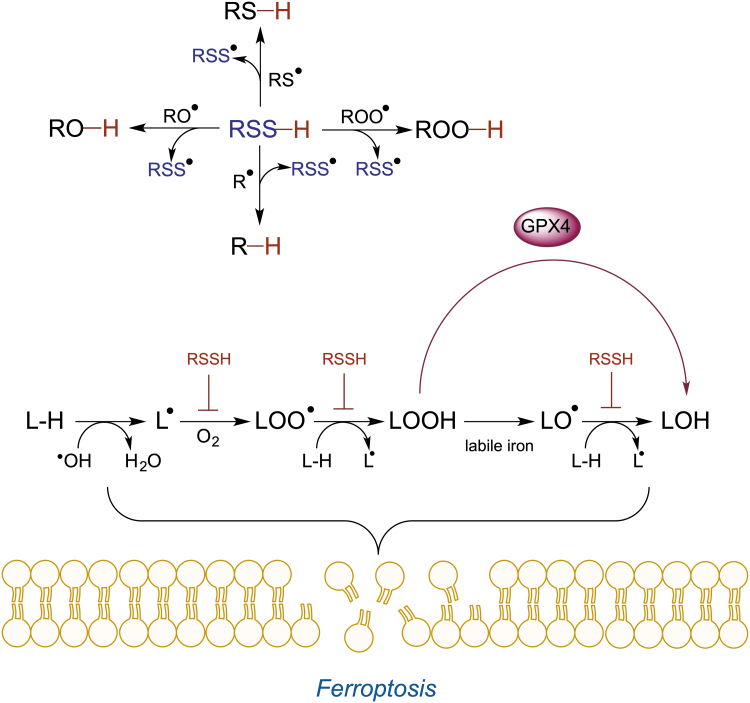
**Antiferroptotic effects of RSSH.** RSSH are excellent H-atom donors (*upper* reaction scheme) preventing formation and radical amplification of lipid radicals (L^•^), peroxyl radical (ROO^•^), and alkoxyl radical (LO^•^), which are the main drivers of membrane damage and ferroptosis (*lower* reaction scheme). GPX4, glutathione peroxidase 4.

The latter has been studied in detail by Pratt's group, where the authors showed that LMW RSSH are excellent H-atom donors and react quite fast with alkyl (*k* ∼ 5 × 10^8^ *M*^−1^ s^−1^), alkoxyl (*k* ∼ 1 × 10^9^ *M*^−1^ s^−1^), peroxyl (*k* ∼ 2 × 10^6^ *M*^−1^ s^−1^), and thiyl (*k* > 1 × 10^10^ *M*^−1^ s^−1^) radicals (Fig. 3) (Chauvin et al., 2017).

The scavenging of alkoxyl and peroxyl lipid radicals is of particular importance, as they lead to cell membrane damage and ferroptotic cell death (Jiang et al., 2021). LMW RSSH efficiently prevented ferroptotic cell death caused by either inhibition or silencing of glutathione peroxidase-4 (Barayeu et al., 2022; Wu et al., 2022). These are exciting observations that demonstrate the pharmacological potential of LMW RSSH. Silencing CSE aggravated lipid peroxidation, while silencing ETHE1 prevented this process, suggesting that endogenously generated LMW RSSH could also act as antiferroptotic agents (Barayeu et al., 2022). This is curious, considering that ETHE1 is located in the mitochondria (Libiad et al., 2014), and so, GSSH accumulation caused by its knockout would be localized in this organelle. Furthermore, ETHE1 silencing causes a devastating disease characterized by cell death (Tiranti et al., 2009).

Keeping in mind the abovementioned chemical characteristics of RSSH, particularly their instability and reactivity with thiols that are present in abundance in the cells, it remains unclear how endogenously generated RSSH could survive long enough to be able to accumulate in and around the lipid bilayer, and even if they do, they would almost exclusively be present in the deprotonated RSS^–^ state (Benchoam et al., 2020; Cuevasanta et al., 2015), which is incompatible with the hydrophobic nature of the cell membrane.

The fate of formed RSS^•^ groups is also of interest. They are relatively stable and do not react with oxygen (Bianco et al., 2016; Chauvin et al., 2016) or nitric oxide (Bianco et al., 2016) (both of which are expected to be more concentrated in the lipid bilayer) (Cuevasanta et al., 2012; Filipovic et al., 2018). If generated in sufficient amounts, RSS^•^ can dimerize to form RSSSSR. Furthermore, biologically relevant electron donors that have sufficient redox potential to reduce RSS^•^ could be vitamin E or vitamin C (0.5 and 0.28 V, respectively) (Buettner, 1993; Everett et al., 1992).

Another biologically important feature of RSSH is their reactivity with biologically important two-electron oxidants, such as H_2_O_2_ and peroxynitrite. Although limited data are available, they all suggest that RSSH react with these species much faster than the corresponding thiols. The reaction of peroxynitrite with albumin persulfide is one order of magnitude faster [(1.2 ± 0.4) × 10^4^ *M*^−1^ s^−1^ at 20°C] than that of the reduced protein (Cuevasanta et al., 2015). Similarly, GSSH reacted 22 times faster with H_2_O_2_ than GSH (Benchoam et al., 2020). Considering the importance that reactive oxygen species (ROS) and reactive nitrogen species have in both the signaling and pathogenesis of many diseases (D'Autréaux and Toledano, 2007; Ferrer-Sueta et al., 2018; Gupta and Carroll, 2014), this “antioxidant” property of RSSH, originally suggested by Paul and Snyder (2012), warrants special attention.

**FIG. 4.**
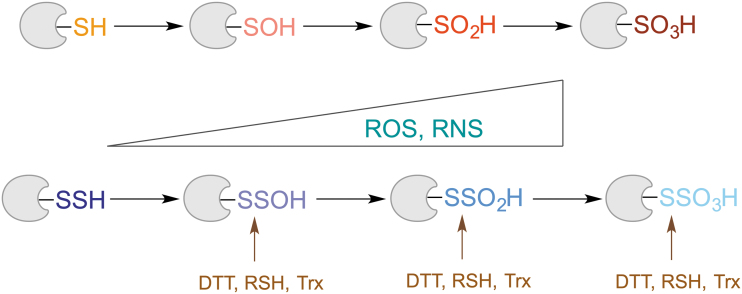
**Reaction of protein persulfides with two-electron oxidants.** DTT, dithiothreitol; RNS, reactive nitrogen species; ROS, reactive oxygen species; Trx, thioredoxin.

Similar to thiols, RSSH react with H_2_O_2_ to form RSSOH, a perthiosulfenic acid (Fig. 4). Computational studies predicted that the reaction of H_2_O_2_ with ethyldisulfane to form the pethiosulfenic species was ∼6.2 kcal/mol more favorable than the oxidation of the corresponding thiol, ethanethiol (Heppner et al., 2018). In the presence of excess oxidant, RSSOH could then undergo further oxidation to perthiosulfinic and perthiosulfonic acids (RSSO_2_H and RSSO_3_H) (Filipovic, 2015; Filipovic and Jovanović, 2017; Ono et al., 2014; Zivanovic et al., 2019). The latter has been observed as an oxidation product while working with different protein persulfides (Cuevasanta et al., 2015; Xiao et al., 2014; Zhang et al., 2014).

Unlike in the case of protein thiols, where oxidation to sulfinic (RSO_2_H) and sulfonic (RSO_3_H) acids is mostly an irreversible process (Akter et al., 2018; Paulsen and Carroll, 2013), due to the S‒S bond present in “perthio” equivalents of these modifications, it is possible to reduce them and restore the thiol (Fig. 4). Indeed, our recent study showed that thioredoxin reacts with cysteine perthiosulfonate ∼2 orders of magnitude faster than it does with cystine (Zivanovic et al., 2019). Nagy's group also confirmed that different thioredoxins (Trx1 and TRP14) could reduce protein-bound perthiosulfonate (Dóka et al., 2020). This is no surprise, as one of the well-known substrates for Trx is the enzyme 3′-phosphoadenosine-5′-phosphosulfate reductase, which forms perthiosulfonate in its active site during the catalytic cycle (Palde and Carroll, 2015).

The existence of this “rescue loop” in which oxidized thiols can be restored back to their reduced state represents the core of the RSSH protective and antiaging hypothesis (Zivanovic et al., 2019) that is discussed in detail later.

## Persulfide Detection

Persulfide detection and quantification have been met with many challenges. The initial reports that 25% of all proteins are in RSSH form seem to be an overestimation (Mustafa et al., 2009). Historically, protein persulfides have been quantified within the total sulfane sulfur pool (Ubuka, 2002; Wood, 1987). Most likely, the most realistic values come from triphenylphosphine-based approaches for sulfane sulfur extraction, as S^0^ is mainly derived from RSSH. As summarized in Table 1, regardless of the final detection method, the reported values in two studies performed 25 years apart are very similar, and they are in the low micromolar range (Hannestad et al., 1989; Liu et al., 2014). Considering that these approaches also detect LMW RSSH, the recent reports of high (100–400 μ*M*) GSSH concentrations are puzzling (Akaike et al., 2017; Ida et al., 2014).

**Table 1.** Protein Sulfane Sulfur Levels Detected by Triphenylphosphine-Based Approach

	Hannestad et al. (1989)	Liu et al. (2014)
Tissue type	nmol/g (μ*M*)^a^	nmol/g (μ*M*)^a^
Plasma	0.4 (0.08)	4.7–13.1 (0.94–2.62)
RBC	0.2 (0.04)	2.3–3.7 (0.46–0.74)
Liver	10.2 (2.04)	57 (11.4)
Kidney	26.8 (5.36)	150.9 (30.18)
Brain	36.7 (7.34)	46 (9.2)
Heart	35.6 (7.12)	61.8 (12.36)
Muscle	6.2 (1.24)	n.d.
Spleen	4.2 (0.84)	56.1 (11.22)

^a^ Cells are typically 10%–20% protein and 60%–75% water. Thus, 1 nmol of sulfane sulfur (mg protein)^−1^ is equivalent to ∼200 μ*M* (Filipovic et al., 2018).

RBC, red blood cell.

In alkaline solutions, persulfides show an absorption maximum at 335–340 nm and a relatively low absorption coefficient (∼310°*M*^−1^ cm^−1^) (Filipovic et al., 2018), but due to the abovementioned reactivity, it has been challenging to selectively detect persulfides in complex mixtures, such as cell lysates. Several methodological approaches have been described for RSSH labeling (Cuevasanta et al., 2015; Mustafa et al., 2009; Sen et al., 2012), but they lacked either specificity (Pan and Carroll, 2013) or sensitivity (Filipovic et al., 2018). A detailed description of these approaches and their limitations can be found elsewhere (Filipovic et al., 2018). In this study, we focus on three methodological approaches that have been recently developed and used for global proteomic mapping of the cellular persulfidome. All three methods rely on the nucleophilic nature of RSSH in solution, and they require an initial reaction with an electrophile, that is, a thiol blocking reagent.

**FIG. 5.**
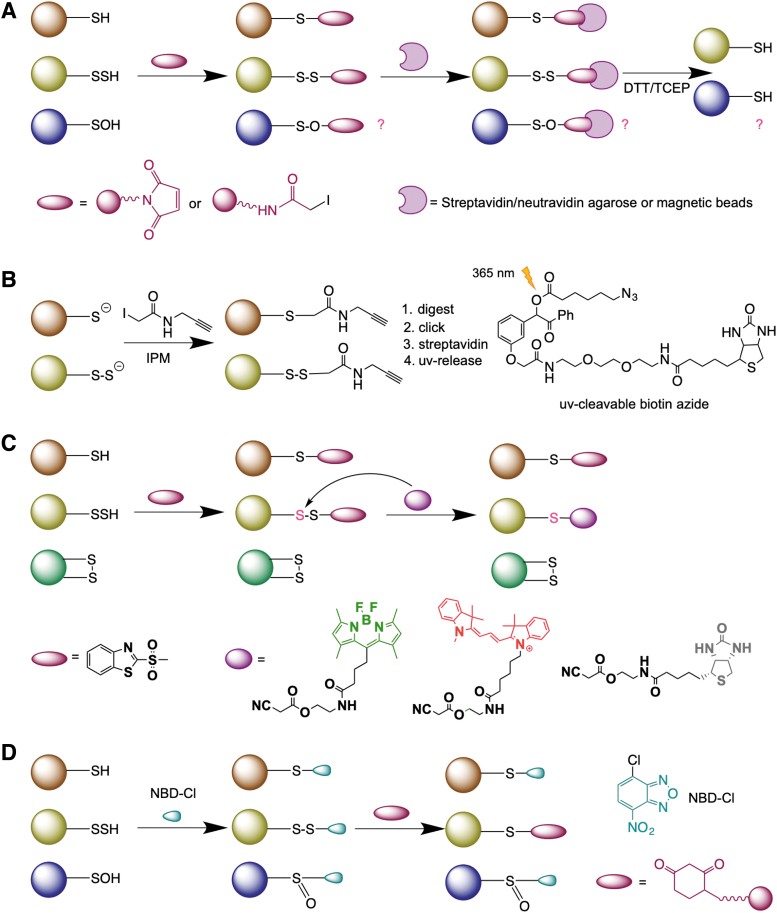
**Methods for persulfide detection. (A)** Biotin thiol assay and its variants. **(B)** Low-ph quantitative thiol reactivity profiling method. **(C)** Tag-switch method. **(D)** Dimedone-switch method.

The biotin thiol assay (BTA) (Gao et al., 2015) and its variants ProPerDP (Dóka et al., 2016) and qPerS-SID (Longen et al., 2016) use a thiol blocking reagent that carries biotin to block both RSH and RSSH, generating thioether in the case of the former and mixed disulfide in the case of the latter (Fig. 5A). Streptavidin enrichment of either proteins or peptides is then performed, and upon treatment with reducing agents (either dithiothreitol [DTT] or tris (2-carboxyethyl) phosphine [TCEP]), a selective release of those that contained an RSSH is achieved (Fig. 5A). However, several concerns have been raised about the selectivity of this approach (Fan et al., 2020). Namely, RSOH also readily reacts with both *N*-ethyl maleimide and iodoacetamide, yielding an adduct that is cleavable with DTT or TCEP (Reisz et al., 2013).

In addition, thioethers formed in the reaction with *N*-ethylmaleimide are also known to be cleaved by DTT *via* the retro-Michael addition (Fontaine et al., 2015). Taking that into consideration, Bibli et al. recently developed a method where cells and tissues were initially lysed in the presence of dimedone to trap all RSOH proteins and then subjected to labeling, and they identified 1536 differentially persulfidated cysteine residues in endothelial cells (Bibli et al., 2021).

Using the same chemical logic, Yang's group recently reported a “direct” method for RSSH detection, low-pH quantitative thiol reactivity profiling (QTRP) (Fu et al., 2020). Alkylation is performed at low pH (5.0), where RSSH are expected to maintain high reactivity (owing to their lower p*K*_a_ and increased nucleophilicity), whereas the majority of free thiols remain protonated and less reactive (Fig. 5B). Using click chemistry and ultraviolet-cleavable biotinylated probes for peptide release (Yang et al., 2015), the authors cleverly avoided the reduction step and directly compared the m/z of peptides, considering the presence of an additional sulfur in the persulfide-containing peptides. A total of 1547 persulfidated sites on 994 proteins were identified in several cell lines (Fu et al., 2020).

We compared their data sets with those generated by BTA (Gao et al., 2015) and qPerS-SID (Longen et al., 2016) and obtained only a minor overlap (82 proteins) between all 3, although the measurements by qPerS-SID and QTRP were performed on the same cell line.

It has been reported recently that the mixed disulfides formed in the reaction of RSSH with commonly used electrophiles (iodoacetamide, *N*-ethylmaleimide, monobromobimane, *etc.*) undergo thiosulfoxide tautomerization and are prone to decomposition in the presence of high amounts of nucleophiles (such as cyanide), leading to the formation of a thioether (Schilling et al., 2022). However, these conditions are not compatible with standard protocols for cell lysis and labeling and should be of concern only when working with isolated proteins. In fact, the same authors recently used the criticized approach to quantify GSSH levels in cell lysates (Barayeu et al., 2022).

Selective labeling of protein persulfides has also been attempted by the tag-switch method (Park et al., 2015; Wedmann et al., 2016; Zhang et al., 2014). This method is based on the premise that thiols and persulfides act as nucleophiles, and so in the first step, a thiol blocking reagent is introduced to tag both P-SH and P-SSH (forming intermediate T) (Fig. 5C). If an appropriate tag is used, the disulfide bonds in the persulfide products will show greatly enhanced reactivity to certain nucleophiles compared with common disulfides in proteins (where there is little difference in the electrophilicity of the two sulfur atoms) (Fig. 5C). Therefore, it is possible to introduce a tag-switching reagent (containing both the nucleophile and a reporting molecule, such as biotin) to label only the persulfide products.

It should be noted that thiol products are thioethers, which are not expected to react with the nucleophile. In the original version of the method, we used methylsulfonyl benzothiazole (MSBT) or its more water-soluble analog (benzothiazole-2-sulfonyl)-acetic acid (MSBT-A) as a thiol-blocking reagent since the mixed aromatic disulfide formed from blocking RSSH showed enhanced reactivity toward cyanoacetic acid-based nucleophiles (Park et al., 2015; Wedmann et al., 2016; Zhang et al., 2014). Aroca et al. (2017) reported >2000 persulfidated proteins in *Arabidopsis thaliana* (∼5% of total proteome) using this approach. The disadvantage of this approach is that MSBT shows limited solubility in water and that cyanoacetate-based probes are unstable under MS conditions, making the analysis very complicated (Aroca et al., 2017; Filipovic et al., 2018).

Recently, we proposed a modification of this approach and named it the dimedone switch method (Zivanovic et al., 2019). MSBT was replaced with 4-chloro-7-nitrobenzofuran (NBD-Cl), a more soluble and well-known thiol blocking reagent (Bernal-Perez et al., 2012; Ellis and Poole, 1997), while the cyanoacetic acid-based probe was replaced by a dimedone-based probe (Fig. 5D) (Klomsiri et al., 2010; Paulsen and Carroll, 2013). Dimedone probes constitute a real advantage since a great number of them are commercially available. Indeed, dimedone is well used for sulfenic acid labeling (Klomsiri et al., 2010; Paulsen and Carroll, 2013). The main challenge was being able to block sulfenic acid and allow dimedone to react with derivatized-persulfides This is one of the advantages of NBD-Cl, which blocks not only RSH and RSSH but also RSOH (Ellis and Poole, 1997).

In addition, NBD-Cl blocks amino groups resulting in green fluorescence, which could be used as a measure of total protein load (Zivanovic et al., 2019). This method has been used to detect persulfides directly in-gel or in cells and whole organisms by microscopy, to perform antibody microarray analysis of persulfidated targets, and for persulfidome analysis by MS (Aroca et al., 2021; Bibli et al., 2021; Pedre et al., 2023; Statzer et al., 2022; Zivanovic et al., 2019).

All methods suffer limitations and will require further improvements. The challenge of selectivity remains the main obstacle.

## Cellular Mechanisms of Persulfide Formation and Removal

To better understand the biological role of RSSH, it is important to understand the cellular mechanisms of their formation. In the last decade, several reaction mechanisms have been shown to lead to intracellular RSSH formation, but further studies are required to better understand how cells produce RSSH.

### Disulfide reduction by H_2_S as a source of protein persulfidation

H_2_S reacts with disulfides to yield RSSH [Eq. (8)] (Cuevasanta et al., 2015; Vasas et al., 2015).


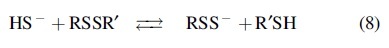


The formation of cysteine persulfide from cystine and H_2_S was reported almost 100 years ago (Andrews, 1926). Thermodynamic calculations suggest that the reaction between HS^−^ and a typical low-molecular disulfide RSSR, such as cystine, to form RSS^−^ and RSH is thermoneutral and has equilibrium constants of ∼1 (Koppenol and Bounds, 2017). Our systematic study of the kinetics of the reactions of H_2_S with low-molecular-weight disulfides and with mixed albumin disulfides (formed between Cys34 of human serum albumin and low-molecular-weight thiols) showed that the reactions are indeed slow (*k*_pH7.4_ = 2–3 *M*^−1^ s^−1^ for cysteinylated or glutathionylated albumin) (Cuevasanta et al., 2015). The rate constants show a clear correlation with the p*K_a_* of the leaving thiol (Cuevasanta et al., 2015).

The slow reaction and relatively low steady-state concentrations of H_2_S suggest that this mechanism probably does not play a major role in protein RSSH formation (Filipovic et al., 2018; Wedmann et al., 2016). However, the specific protein environment could make some cysteines more acidic than others. Prxs are a good example, with the p*K_a_* of the active-site cysteine being below 6 (Wood et al., 2003). Indeed, in persulfidome analysis, two Prxs from Aspergillus fumigatus (Aspf3 and PrxA) were found to be persulfidated, and the presence of their disulfide form increased in cells lacking H_2_S-producing enzymes (Sueiro-Olivares et al., 2021).

### Metal-catalyzed protein persulfidation *via* radical formation

Redox-active metal centers, particularly in iron heme proteins, are known to react with sulfide, resulting in its oxidation to different reactive sulfur species (Bostelaar et al., 2016; Pálinkás et al., 2015; Ruetz et al., 2017; Vitvitsky et al., 2015). One such protein is cytochrome c (Cyt C). Heme in Cyt C is more exposed, and the product of H_2_S oxidation can easily be reached by the surrounding proteins, resulting in protein persulfidation (Alvarez-Paggi et al., 2017). Reaction between Cyt C and H_2_S results in the initial formation of an HS^•^/S^•−^ radical (Vitvitsky et al., 2018; Wedmann et al., 2014) that can react with proteins to yield an RSSH [Eqs. (9) and (10)].

(9) $$HS^{∙}+RS^{-}\to RSSH^{∙-}$$

(10) $$RSSH^{∙-}+O_{2}\to RSSH+{O_{2}}^{∙-}$$

**FIG. 6.**
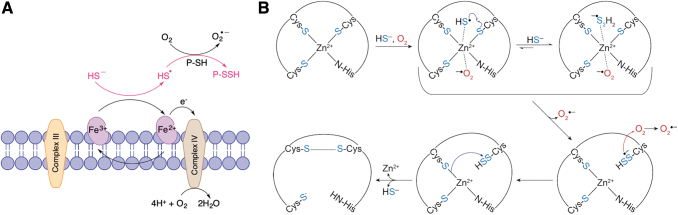
**Metalloprotein-catalyzed RSSH formation. (A)** Cytochrome c oxidizes H_2_S to HS^•^, which can react with protein thiols to form RSSH. Reduced cytochrome c is then reoxidized by complex IV of mitochondrial respiratory chain, establishing a pseudocatalytic cycle for RSSH formation. **(B)** Zinc center in metalloproteins could play a general role in catalyzing protein persulfide formation by: (i) shifting the redox potential of O_2_ to a more positive value favoring superoxide formation, (ii) lowering the p*K*_a_ of H_2_S (akin to Zn binding to OH^−^ in carbonic anhydrase) favoring formation of HS^•^, and (iii) acting as a template to bring O_2_ and H_2_S in a close proximity enabling efficient electron shuttling.

As reduced Cyt C passes on electrons to complex IV of the mitochondrial respiratory chain, it reoxidizes, establishing a pseudocatalytic cycle for HS^•^/S^•−^ radical generation and mitochondrial protein persulfidation (Fig. 6A). We observed that H_2_S reacts with Cyt C, leading to persulfidation of various targets (Vitvitsky et al., 2018). Silencing of Cyt C resulted in a profound decrease in protein persulfidation caused by mitochondria-targeted H_2_S delivery (Vitvitsky et al., 2018). During the initiation of apoptosis, Cyt C leaks out of mitochondria putting it in proximity of procaspase 9 (Riedl and Salvesen, 2007). We reported that Cyt C catalyzes procaspase 9 persulfidation, inhibiting apoptosis (Vitvitsky et al., 2018).

Far more intriguing was the observation that H_2_S can interact with zinc finger proteins causing protein persulfidation (Lange et al., 2019). Zinc is not a redox-active metal, and so, no reaction other than potential coordination is expected. However, exposing the zinc finger protein tristetraprolin to H_2_S in air resulted in persulfide formation, something that could not be achieved under anaerobic conditions. We proved that Zn^2+^ would coordinate HS^−^ and serve as a catalyst for electron shuttling from HS^−^ to O_2_, resulting in the formation of HS^•^/S^•−^ and superoxide anion (Fig. 6B) (Lange et al., 2019). This mechanism may not only be characteristic for this particular protein. Indeed, analysis of recently published persulfidome data sets (Fu et al., 2020) shows that a significant portion of identified proteins (104/994) contains zinc.

### Reaction of RSOH with H_2_S

Sulfenylated cysteines are products of the reaction of H_2_O_2_ with RSH, and they play an important role in H_2_O_2_-based redox signaling (Gupta and Carroll, 2014; Paulsen and Carroll, 2013; Yang et al., 2014). Early attempts to generate protein persulfides showed that the treatment of proteins with H_2_O_2_ and H_2_S resulted in RSSH formation [Eq. (11)] (Ida et al., 2014; Zhang et al., 2014).

(11) $$HS^{-}+RSOH\to RSSH+OH^{-}$$

RSOH of human serum albumin reacted ∼600 times faster with H_2_S than with GSH at pH 7.4, while the intracellular RSSH levels increased upon treatment with H_2_O_2_ in a manner that is dependent on the activity of H_2_S-producing enzymes (Cuevasanta et al., 2015; Wedmann et al., 2016).

Recent attempts to understand the importance of this reaction showed that cells lacking CSE undergo massive sulfenylation when exposed to H_2_O_2_ concentrations that do not change the RSOH status in wild-type cells. Treatment with an H_2_S donor abrogated this effect, suggesting that RSOH to RSSH conversion may be the main manner of sulfenylation resolution in some cases (Zivanovic et al., 2019).

**FIG. 7.**
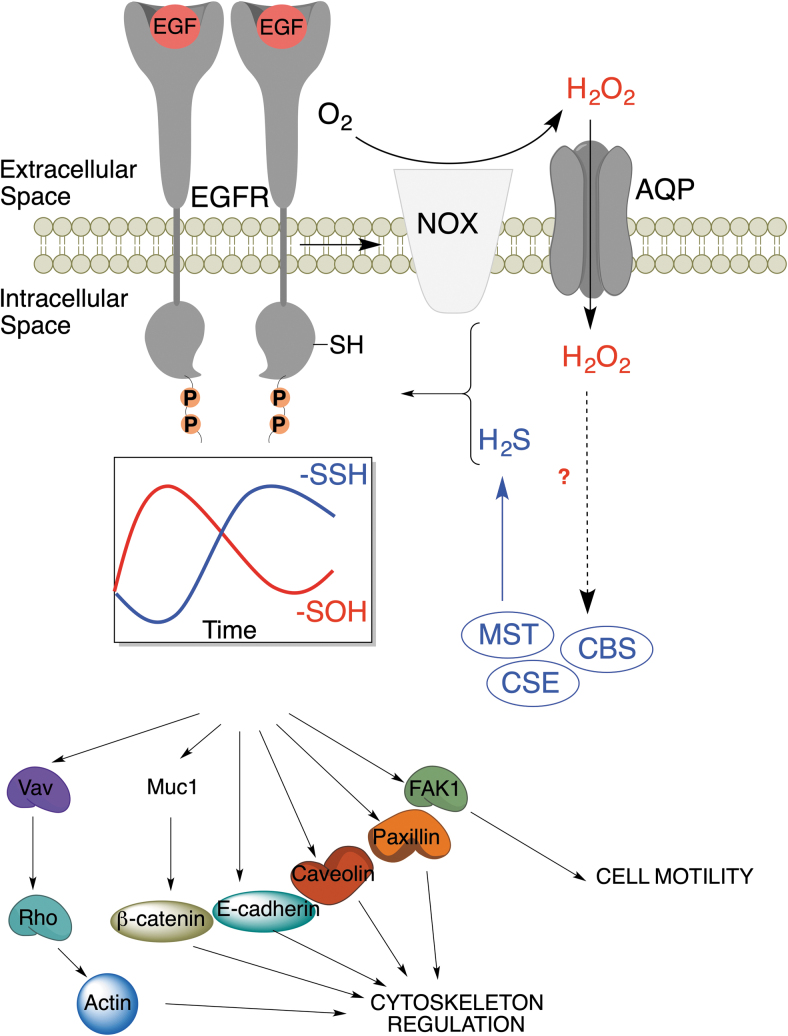
**Regulation of EGFR signaling by RSOH to RSSH switching.** Upon the receptor stimulation with EGF, NOX produces H_2_O_2_, which is transported into the cell *via* aquaporins, and H_2_O_2_ modifies C797 of EGFR to RSOH, increasing its kinase activity. H_2_O_2_ also stimulates expression of H_2_S producing enzymes. H_2_S reacts with RSOH to form RSSH on EGFR, which inhibits the phosphorylation of EGFR and downstream signaling. The temporal phase shifted waves of RSOH and RSSH caused by EGFR activation modulate many of the downstream targets involved in cytoskeleton regulation and cell motility. EGFR, epidermal growth factor receptor; H_2_O_2_, hydrogen peroxide; NOX, NADPH oxidase.

A good example is signaling by receptor tyrosine kinases, which is intrinsically linked to H_2_O_2_ formation (Paulsen and Carroll, 2013; Paulsen et al., 2012; Sundaresan et al., 1995). By treating the cells with epidermal growth factor (EGF), vascular epithelial growth factor, or insulin, we observed that the initial wave of RSOH formation (caused by the activation of the corresponding receptors and subsequent H_2_O_2_ generation) was followed by a wave of persulfidation in a phase-shifted manner. Manipulation of H_2_S generation affected both the amplitude and the duration of the phase, leading to the conclusion that the RSH to RSOH to RSSH transformation represents an inherent thiol redox switch (Fig. 7).

Redox switching can alter protein structure and function (D'Autréaux and Toledano, 2007). The EGF receptor (EGFR) undergoes RSH to RSOH transformation at C797 upon activation with EGF, which enhances its kinase activity (Paulsen et al., 2012). We observed that EGFR stimulation increases the expression of MPST, CSE, and CBS to produce more H_2_S, resulting in the RSOH to RSSH transformation. This switch inhibits kinase activity, as demonstrated by decreased receptor activity and Y1068 phosphorylation in cells treated with H_2_S donors (Zivanovic et al., 2019). Furthermore, EGFR downstream targets have also been found to be persulfidated upon receptor activation (Fig. 7).

### Enzymatic production of LMW RSSH

The pyridoxal-phosphate enzymes CBS and CSE can, in addition to their canonical reactions, use cystine and, in the case of CSE, homocystine and cysteine-homocysteine disulfide, to form the corresponding persulfides (Ida et al., 2014; Yadav et al., 2016). Work from Akaike's group originally suggested that CSE and CBS could serve as the main sources of cysteine persulfide in cells (Ida et al., 2014). However, simulation of cysteine persulfide and homocysteine persulfide formation from CSE and CBS indicated that cysteine persulfide formation by CSE and CBS is very low and that homocysteine persulfide synthesis by CSE is negligible under intracellularly available substrate concentrations (Yadav et al., 2016). Nonetheless, under specific pathological conditions that could result in increased cystine concentrations, this reaction could become an important source of LMW RSSH.

CARS is an enzyme that produces Cys-tRNA *via* cysteine and aminoacyl-tRNA (Fujii et al., 2019), but CARS also has a moonlighting function as a cysteine persulfide synthase in a manner that is independent of the aminoacyltransferase reaction (Akaike et al., 2017). LMW RSSH levels were also lower in mitochondria from CARS2 knockout mice. In addition to cysteine persulfide synthase activity, the authors also proposed that CARS2 could use cysteine persulfide as a substrate, making Cys-SSH-tRNA, which could eventually be integrated into proteins (Akaike et al., 2017).

SQR remains the main source of LMW RSSH. Localized in the mitochondrial inner membrane, SQR is a member of the flavin disulfide reductase superfamily involved in H_2_S detoxification (Filipovic et al., 2018; Landry et al., 2019; Mishanina et al., 2015). Recent crystal structure analysis of human SQR revealed that the enzyme's active-site resting state is in the form of a trisulfide (Landry et al., 2019). H_2_S reacts with it, forming RSSH at C201, which forms an intense charge-transfer complex with flavin adenine dinucleotide and persulfide formation at C379, which transfers sulfur to an external acceptor. Both GSH and CoA can serve as the external acceptor, resulting in efficient formation of GSSH and CoA-SSH, respectively (Landry et al., 2019).

The electrons released in this process are transferred to coenzyme Q, which feeds them into the mitochondrial respiratory chain, leading to the proposal that H_2_S is an inorganic substrate for mammalian respiration (Goubern et al., 2007). It is worth mentioning that both MPST and TST could also be involved in the formation of LMW RSSH, particularly GSSH (Filipovic et al., 2018; Mishanina et al., 2015; Yadav et al., 2013).

### Transpersulfidation

**FIG. 8.**
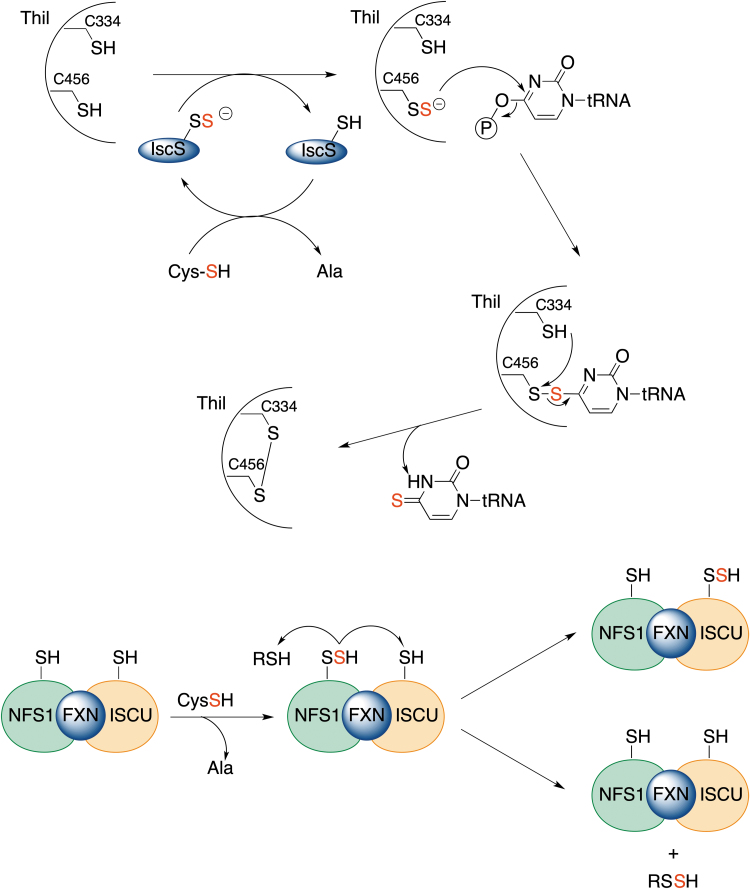
**Transpersulfidation steps in RNA thiolation and iron–sulfur cluster assembly.** Cysteine desulfurase (IscS in prokaryotes, NFS1 in eukaryotes) converts cysteine to alanine forming the intermediate persulfide (sulfur originating from the cysteine is marked *red*). Through transpersulfidation, this sulfur is transferred to ThiI and then to tRNA (*upper* figure), or transferred to ISCU. Alternatively, NFS1 persulfide could transfer sulfur to LMW RSH. FXN, frataxin, a protein involved in iron–sulfur cluster assembly; ISCU, iron-sulfur cluster assembly scaffold protein; LMW, low molecular weight; ThiI, tRNA sulfur transferase.

The transfer of sulfane sulfur has emerged evolutionarily as a method to allow iron–sulfur cluster assembly or regulate transcription through thionucleoside generation on tRNA (Fig. 8) (Kessler, 2006; Lill and Mühlenhoff, 2005). All these processes involve protein persulfides, and sulfane sulfur is transferred from one protein to another *via* transpersulfidation. The process starts with cysteine desulfurase, NFS, a PLP-dependent enzyme, which converts cysteine to alanine, generating protein persulfide (Fig. 8) (Mueller, 2006; Zhang et al., 2010). On the way to the iron–sulfur cluster assembly, this persulfide is then transferred further to other protein targets, as well as LMW thiols (Parent et al., 2015). In addition, the cysteine desulfurase persulfide shuttles the sulfhydryl sulfur to a rhodanese-like sulfur transferase, ThiI, forming ThiI persulfide. ThiI persulfide then uses the sulfur for synthesis of the 2-thiouridine modification in tRNA (Fig. 8).

The enzymes involved in sulfur transfer share homology with the rhodanese domain fold with an α/β topology, which can be present in a single copy, in tandem repeats or fused with other domains (Bordo, 2002; Libiad et al., 2015). In fact, Bonomi et al. (1977a) and Bonomi et al. (1977b) observed that rhodanese is capable of transferring sulfane sulfur to protein targets, such as succinate dehydrogenase, yeast alcohol dehydrogenase, and bovine serum albumin. MPST is also known to be able to transfer sulfur from its substrate, 3-mercaptopyruvate, to the iron–sulfur chromophore of adrenal ferredoxin, similar to rhodanese (Taniguchi and Kimura, 1974). Thiosulfate sulfur transferase-like domain-containing 1 protein (TSTD1) has been recently identified (Libiad et al., 2018).

**FIG. 9.**
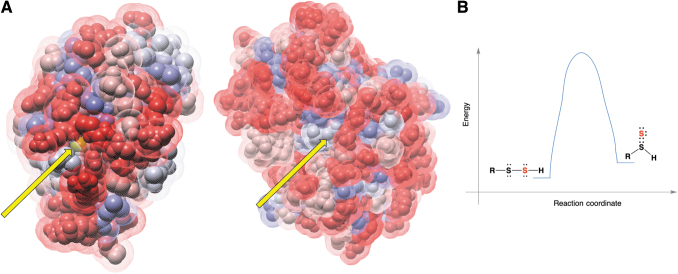
**Protein transpersulfidation. (A)** Cysteine persulfide of TSTD1 (*left*) is more exposed (*yellow arrow*) and accessible to engage in transpersulfidation reactions than cysteine of TST (*right*). **(B)** Energy diagram of RSSH tautomerization to thiosulfoxide (sulfane sulfur is marked *red*). TSTD1, thiosulfate sulfur transferase-like domain-containing 1 protein.

The protein structure shows an active site that is quite exposed and distinct from that of rhodanese and MPST (Fig. 9A). TSTD1 can readily transfer sulfur to Trx, forming thioredoxin persulfide. The other potential protein targets of TSTD1 remain unclear (Libiad et al., 2018).

Transpersulfidation in these proteins occurs only because of the structure of the active site that surrounds the reacting cysteine. For example, C247 of rhodanese is located at the intersection of the axes of two helices, which contribute a significant electrical field and lower the p*K*_a_ of the thiol group (estimated to be 6.5) by ∼3.5 units, making this sulfur atom very nucleophilic. The terminal sulfur atom in the persulfidated protein interacts with the positively charged side groups of Arg186 and Lys249, which masks the negative charge of the persulfide, making this sulfur atom less nucleophilic than it would be in a nonprotein environment (Ploegman et al., 1979; Ploegman et al., 1978). Therefore, the setting of the active site favors nucleophilic attachment at the terminal sulfur, that is, it favors transpersulfidation.

LMW RSSH are not expected to get involved in the transpersulfidation reaction; formation of a mixed disulfide and the release of H_2_S are preferred (Fig. 2A). For transpersulfidation to happen, an RSSH would have to undergo tautomerization to its thiosulfoxide form [RS(H) = S] (Steudel et al., 1997). Computational studies and bond energies suggest that although thiosulfoxide tautomers are only 5 kJ/mol less stable than the corresponding disulfanes, they cannot be formed, as the energy barrier for isomerization is >100 kJ/mol (Steudel et al., 1997) (Fig. 9B).

In addition to sulfur transferases, the rhodanese homology domain is present in phosphatases of the Cdc25 family (involved in regulation of the cell cycle), phosphatases of the mitogen-activated protein kinase family, several ubiquitin hydrolases, and heat shock, cold shock, and phage shock proteins (Bordo, 2002). It is tempting to speculate that some of those proteins could also serve as transpersulfidases, providing the context for some specificity of RSSH formation. Recently, Pedre et al. (2023) suggested that MPST could be the main cellular persulfidase. They showed that expression of *Saccharomyces cerevisiae* ortholog of MPST, TUM1, increases protein persulfidation in mammalian cells, which contrasts with the observation of Zivanovic et al. (2019), who showed no change in RSSH levels in Δ*tum-1* mutants of *S. cerevisiae*. Knockout of MPST resulted in downregulation of protein persulfidation of only 64 proteins.

For comparison, the CSE knockout results in reduced persulfidation of 188 proteins (Bibli et al., 2021). It remains to be elucidated how MPST interacts with the identified targets (some of which do not contain surface-exposed cysteine) and how it transfers the sulfane sulfur.

### Depersulfidation

**FIG. 10.**
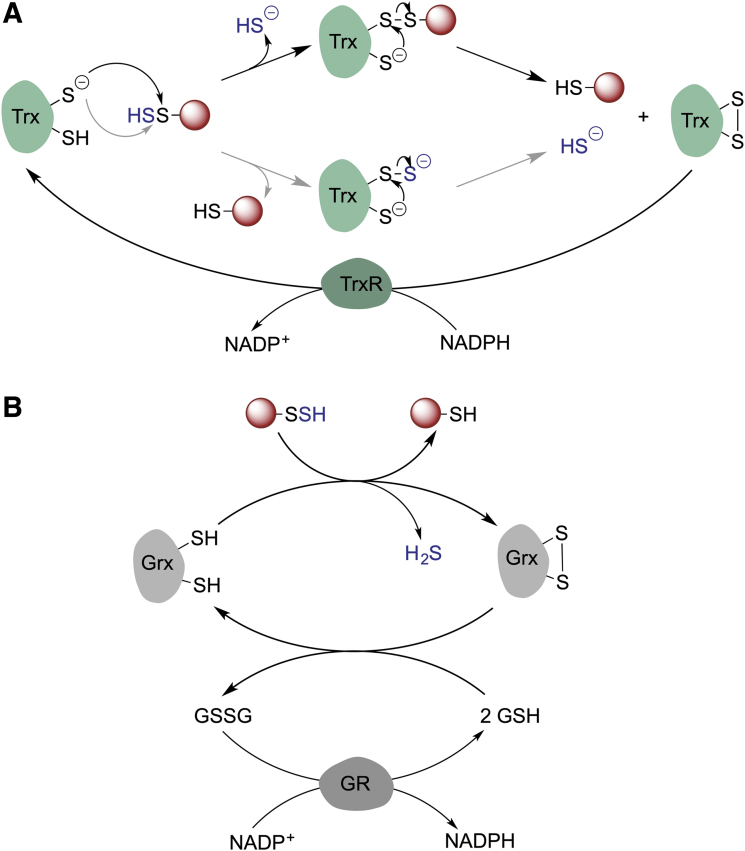
**Enzyme-catalyzed protein depersulfidation. (A)** Trx catalyzes protein depersulfidation *via* two different mechanisms. Oxidized Trx is then reduced by TrxR. **(B)** Grx catalyzes protein depersulfidation oxidizing GSH, which is reduced back by GR. GR, glutathione reductase; Grx, glutaredoxin; TrxR, thioredoxin reductase.

For persulfidation to have a regulatory function, cells should have mechanism(s) to remove persulfidation, that is, depersulfidase proteins, and restore reduced thiols. In cells, Trx, a disulfide oxidoreductase, serves as a main redox partner of a variety of client proteins. Trx performs disulfide reduction in conjunction with thioredoxin reductase (TrxR) (Buchanan et al., 2012; Lu and Holmgren, 2014b). As protein persulfides are analogous to disulfides, the Trx/TrxR system seems to be a good candidate for protein depersulfidation. Trx is involved in H_2_S release from MPST (Yadav et al., 2013), and it was ∼200 times more efficient than DTT in reducing persulfidated phosphatase PTP1B (Krishnan et al., 2011). Nagy's group (Dóka et al., 2016) and our group (Wedmann et al., 2016) observed that the Trx/TrxR system controls intracellular persulfidation globally (Fig. 10A).

When total intracellular persulfidation levels were assessed as a function of lysis time, the levels notably decreased. The addition of auranofin, an inhibitor of the Trx/TrxR system, to the lysis buffer normalized these levels, keeping the persulfidation constant (Wedmann et al., 2016). Trx efficiently reduced both LMW and protein persulfides, leading to H_2_S release. The first-order rate constant for the reaction of Trx with cysteine persulfide was estimated to be 4.5 ± 0.1 *M*^−1^ s^−1^, which is almost one order of magnitude higher than that for cystine. A similar rate constant was observed for HSA-SSH (4.1 ± 0.8 *M*^−1^ s^−1^) (Wedmann et al., 2016). Lower circulatory sulfane sulfur levels were observed in HIV patients with a high viral load and high circulatory Trx levels than in patients treated with antiretroviral therapy, supporting that Trx has depersulfidase activity even in humans (Wedmann et al., 2016).

Two mechanisms are possible to explain the reaction: (i) transfer of the outer sulfur from the persulfide to the catalytic cysteine of Trx leading to the transient formation of Trx-SSH, which would then react with the resolving cysteine of Trx, resulting in a displacement of HS- and formation of an intramolecular disulfide bond in Trx, or (ii) a nucleophilic attack of one of the Trx cysteines to the inner sulfur of the persulfide with immediate elimination of H_2_S and formation of a mixed Trx-client disulfide complex, followed by the displacement of the client thiol by the resolving cysteine and formation of a disulfide bond in Trx (Fig. 10A). While the latter mechanism is used to explain the disulfide reductase activity of Trx, in the case of depersulfidase activity, either mechanism could result in H_2_S generation (Libiad et al., 2018).

A thioredoxin-related protein of 14 kDa (TRP14) could also play an important role in depersulfidation, as its knockdown led to an increase in protein persulfidation (Dóka et al., 2016). TRP14 is particularly interesting since it may become the main depersulfidase under conditions of oxidative stress when Trx becomes heavily engaged in turning over the Prx system (Dóka et al., 2016).

Nagy et al. (2014) also addressed the role of the GSH system (glutathione reductase [GR], GSH, and glutaredoxin [Grx]) in catalyzing depersulfidation. The overall structure of GR is similar to that of TrxR and conserved throughout all kingdoms. GR is also an FAD-containing disulfide oxidoreductase that uses NADPH as a source of electrons, but unlike TrxR, GR does not have selenocysteine in its active site (Fig. 10B) (Lu and Holmgren, 2014a). In the presence of GSH and NADPH, GR efficiently reduced polysulfides and BSA-SSH *in vitro* and at higher rates when Grx was introduced (Dóka et al., 2016).

The removal of protein persulfides seems to be intrinsically linked to the availability of NADPH as a reducing power. Future studies should address how metabolic changes in the NADPH/NADP^+^ ratio control RSSH levels of specific proteins.

## Persulfidation Affects Protein Function

Despite the large number of proteins found in different proteomics screens, there are only a few examples where a clear correlation between persulfidation and function is established. We divided those into examples showing the gain of enzymatic activity or the loss of enzymatic activity.

### Gain of enzymatic activity

GAPDH was the first protein characterized as persulfidated in a study that sparked this whole research field (Mustafa et al., 2009). GAPDH is an important glycolytic enzyme but is also known as a regulator of the cell death cascade (Hara et al., 2005). Snyder's group showed that GAPDH is persulfidated at C152, and this modification increases its enzymatic activity approximately sevenfold. DTT treatment of GAPDH decreases its activity, suggesting that endogenous persulfidation regulates its function. CSE knockout mice showed ∼35% reduced activity of GAPDH compared with control mice (Mustafa et al., 2009). Under conditions of endoplasmic reticulum stress, where H_2_S production increases, the activity of GAPDH increased as well, while the total amount of protein remained the same, suggesting some sort of posttranslational activation of the enzyme (Gao et al., 2015).

Cotreatment of GAPDH with H_2_O_2_ and H_2_S results in persulfidation of C152 and an increase in enzymatic activity, further supporting the original claims (Gao et al., 2015).

However, Jarosz et al. (2015) worked with purified GAPDH to show that treatment with polysulfides in fact results in inhibition of enzyme activity (∼42% compared with the untreated enzyme) (Jarosz et al., 2015). The inhibition was due to the persulfidation of cysteine residues out of the active site, that is, C156 and C247. Furthermore, when they used the C156S mutant, they observed additional persulfidation at C152 in GAPDH treated with polysulfides, but the enzyme was still inhibited. However, the mutant was also modified at C247 (Jarosz et al., 2015). The identification of C156 and C247, but not C152, as targets of persulfidation was recently confirmed by proteomic studies as well (Fu et al., 2020). More detailed studies addressing the actual conformational effect of RSSH on specific cysteine residues could help resolve these conflicting observations.

**FIG. 11.**
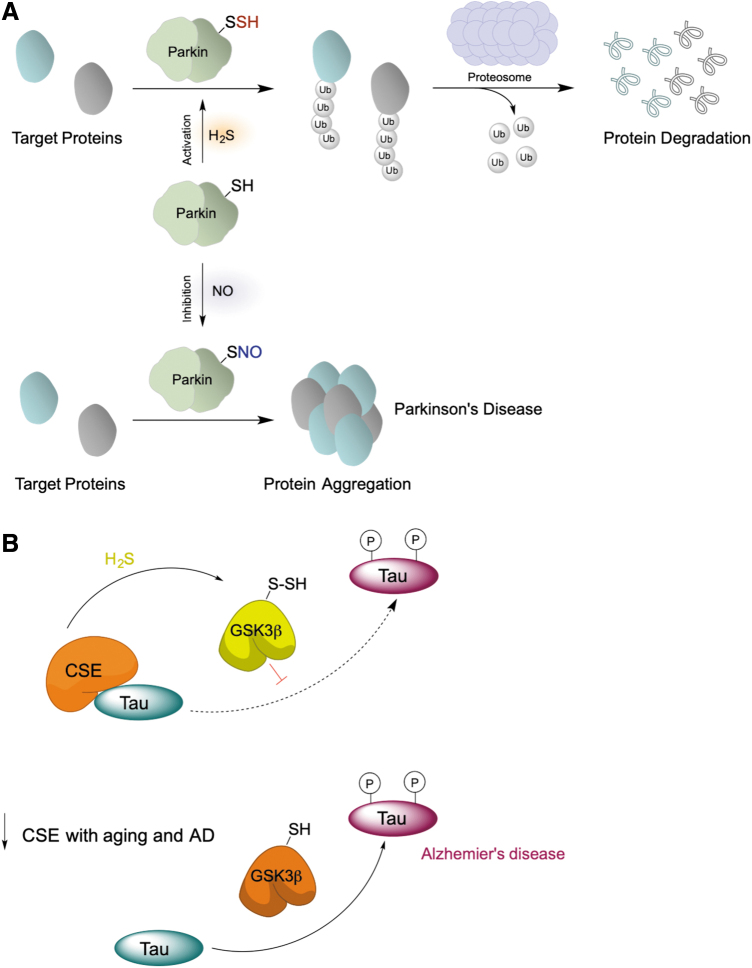
**Persulfidation affects enzyme activity**. **(A)** Persulfidation of parkin stimulates its E3 ligase activity leading to higher ubiquitination of target proteins and their proteosomal degradation. In Parkinson's disease, H_2_S production declines and nitrosation of thiols occurs, leading to inactivation of parkin. **(B)** Tau interacts with CSE stimulating its H_2_S producing activity, which results in persulfidation of GSK3β and inhibition of its activity. With aging and in AD, there is a decline in CSE levels. GSK3β becomes more active leading to hyperphosphorylation of tau and its aggregation. AD, Alzheimer's disease; GSK3β, glycogen synthase kinase 3β.

Similar to GAPDH, persulfidation of parkin, an E3 ligase implicated in Parkinson's disease (Chung et al., 2004), increased its enzymatic activity (Fig. 11A) and rescued neurons from cell death by removing damaged proteins (Vandiver et al., 2013). More importantly, markedly decreased parkin persulfidation has been found in Parkinson's disease human brains, whereas S-nitrosation was increased (Vandiver et al., 2013).

### Loss of enzymatic function

ATG18a is a core autophagy protein in *A. thaliana* that binds to phosphoinositides (Dove et al., 2004; Wun et al., 2020). It forms a complex with ATG2, leading to autophagosome formation. This protein is of particular importance for the induction of autophagy under abiotic stress. Recently, Aroca et al. (2021) showed that persulfidation of C103 in ATG18a inhibits autophagy under endoplasmic reticulum stress conditions by regulating the number and size of autophagosomes. The C103 residue is located inside a hydrophobic cavity formed by ^83^FNQD^86^ and F90 amino acids that have also been found in human orthologs of this protein. Through electrostatic interactions, positive charges around this region strengthen the binding of negatively charged phosphatidylinositide molecules. Persulfidation of C103 results in the introduction of a bulky sulfur that alters the cavity size, disturbing the existing hydrophobic interactions and enforcing new ones.

The negative charge that RSH to RSSH replacement introduces into this hydrophobic cavity due to the lower p*K*_a_ of persulfide further affects the size of the cavity, hindering the interaction with additional phosphoinositide molecules (Aroca et al., 2021).

DJ-1 (also called PARK7) is a ubiquitously expressed protein associated with autosomal recessive early-onset Parkinson's disease (Bonifati et al., 2003). The protein has glyoxalase and deglycase enzymatic functions, but also serves as a redox sensor and a buffer for reactive species (Canet-Avilés et al., 2004; Kinumi et al., 2004; Oh and Mouradian, 2017). C106 was shown to undergo hyperoxidation to RSO_2_H and RSO_3_H. The former is known to translocate to the mitochondria, where it protects cells against cell death (Canet-Avilés et al., 2004). We found DJ-1 to be endogenously persulfidated in human red blood cells, which prompted us to test the hypothesis of protective waves of persulfidation, since chemical tools to detect all thiol oxidation states (RSH, RSOH, RSSH, RSO_2_H, and RSO_3_H) exist (Zivanovic et al., 2019).

Indeed, mouse embryonic fibroblasts exposed to H_2_O_2_ show a protective wave of persulfidation of DJ-1, while cells lacking CSE show pronounced hyperoxidation of C106 (Zivanovic et al., 2019). Recently, Galardon et al. (2022) studied the effect that persulfidation can have on DJ-1 structure and activity. Although both RSSH and RSO_2_H formations led to inhibition of enzymatic function, the structural analysis showed quite distinct conformational changes. Crucial structures implicated in the stabilization of reduced dimers are either lost or weakened in persulfidated proteins. Furthermore, C106 becomes more accessible after persulfidation, making it an easy target for the depersulfidase activity of Trx/TrxR, which would restore the RSH form of C106 (Galardon et al., 2022).

Another example where RSSH exhibits an inhibitory effect is glycogen synthase kinase 3β (GSK3β) (Giovinazzo et al., 2021). GSK3β is one of the busiest kinases in the cell, with >100 identified substrates (Beurel et al., 2015). In Alzheimer's disease (AD), GSK3β phosphorylates tau protein (MAPT), leading to its dissociation from microtubules and increasing its propensity to aggregate, which results in the formation of neurofibrillary tangles, one of the most prominent features of the disease (Crews and Masliah, 2010; Hooper et al., 2008; Lauretti et al., 2020). Recently, we showed that GSK3β could be persulfidated and that its persulfidation levels (as well as global persulfidation levels) decreased in cells, mouse and human brain samples of AD patients (Giovinazzo et al., 2021). MS analysis identified C218 as a target of persulfidation.

C218 is located very close to the active site, E181, so insertion of a bulky sulfur carrying a negative charge would conformationally change the active site, leading to the observed loss of kinase activity (Giovinazzo et al., 2021). We postulated that persulfidation of GSK3β would have protective effects against AD due to its role in inhibiting the enzyme and that loss of persulfidation caused by the decline of CSE levels due to age and disease would result in higher kinase activity and tau hyperphosphorylation (Fig. 11B).

## Future Directions: Protection *Versus* Signaling

In addition to signaling effects, protein persulfidation could have a general protective effect. During oxidative stress, cysteine residues are oxidized to sulfenic acids. This represents an important signaling event for the cell to either start proliferating or to die (depending on the amount of H_2_O_2_) (Lo Conte and Carroll, 2013; Paulsen and Carroll, 2013). If buried deep into protein pockets, an RSOH could become stabilized and not easily reachable for reduction (Paulsen and Carroll, 2013; Shi and Carroll, 2020). H_2_S is small and can reach deep into the protein structure to lead to RSSH formation. Once formed, an RSSH can be reduced back to a thiol by the Trx system (Dóka et al., 2016; Wedmann et al., 2016).

**FIG. 12.**
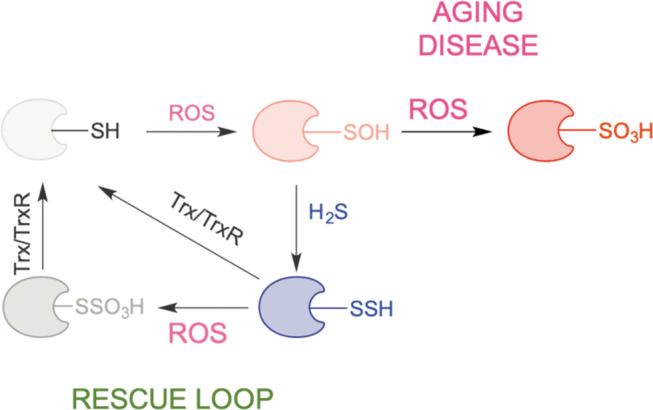
**Hypothetic scheme for antiaging effects of RSSH.** During aging or age-induced diseases, restoring protein persulfidation can serve as a mechanism to reduce ROS-oxidized cysteine residues, preventing their irreversible overoxidation.

When oxidative stress persists (such as in aging and many ROS-related diseases), sulfenylated cysteines oxidize further to RSO_2_H and RSO_3_H (Chauvin and Pratt, 2017), oxidations that are generally considered irreversible (although some RSO_2_H groups can be reduced back to thiols) (Akter et al., 2018). Persulfidated residues are expected to act as better scavengers of ROS than regular thiols, resulting in the formation of RSSO_3_H. The existence of the S‒S bond makes this species a potential target for Trx and the restoration of native thiolate *via* this rescue loop (Fig. 12) (Dóka et al., 2020; Zivanovic et al., 2019). Thus, the overall structure, function, and half-life of thiol-containing proteins are preserved.

We proposed that this recue loop exemplifies a remnant of times when life emerged in an H_2_S-rich environment and that it represents the simplest way to resolve cysteine oxidation and protect proteins from oxidative damage (Petrovic et al., 2021; Zivanovic et al., 2019). Indeed, persulfidation is evolutionarily conserved, and its levels are directly correlated with the organismal ability to fight oxidative stress (Zivanovic et al., 2019).

When life emerged in an H_2_S-rich environment, despite the absence of oxygen, living systems were exposed to high amounts of ROS formed by photolysis of water and metal-catalyzed processes (He et al., 2021). The described rescue loop would have saved the proteins from oxidative damage and extended their “half-life” and their function, playing a role in successful maintenance of life itself. However, a decrease in the expression of H_2_S producing enzyme (primarily CSE, but also CBS and MPST) and consequently the loss of persulfidation were found in aging and different age-induced diseases, such as neurodegenerative diseases (Giovinazzo et al., 2021; Petrovic et al., 2021; Snijder et al., 2015; Statzer et al., 2022; Zivanovic et al., 2019).

This led us to establish the hypothesis of evolutionarily conserved, protective antiaging effects of protein persulfidation (Zivanovic et al., 2019). It is not surprising then that one of the genes affected in many different life span extension interventions is actually CSE (Tyshkovskiy et al., 2019). Furthermore, several studies have shown that dietary interventions, such as calorie restriction, lead to increased H_2_S formation and higher RSSH levels *via* CSE upregulation (Bithi et al., 2021; Hine et al., 2015; Statzer et al., 2022; Zivanovic et al., 2019).

Due to the absence of a clear protein-catalyzed mechanism of RSSH formation and only a dozen examples with clear functional outcomes, how much protein persulfidation is indeed functional is unclear (Hanna et al., 2023). In the eyes of a biologist, this process might appear stochastic and therefore can be disregarded as a nonsignaling process; however, that presumption is inaccurate. As mentioned, RSO_x_H is mainly an irreversible modification that leads to inactivation and possibly acts as a target for protein degradation. Even if the RSSH modification had the same inhibitory effects on the enzyme's function, this inactivation would be reversible and therefore short-lived. The best examples are the abovementioned DJ-1 and the protein tyrosine phosphatase PTP1B. It has been shown recently that the reversibility of the RSSO_3_H modification on PTP1B represents a way that signaling pathways controlled by this phosphatase are regulated (Dóka et al., 2020).

Furthermore, it seems that many proteins found to be persulfidated are structural proteins (Bibli et al., 2021; Fu et al., 2020), which raises the question of how the RSSH modification affects their role. Recent advances in cell biology suggest that the regulation of cellular function could be achieved through a biophysical phenomenon called liquid‒liquid phase separation (LLPS) (Banani et al., 2017; Brangwynne et al., 2009; Hyman et al., 2014; Lafontaine et al., 2021; Shin and Brangwynne, 2017). LLPS is a dynamic and reversible process through which biomolecules, such as proteins or nucleic acids, associate and form membrane-less structures, adding another layer of complexity to the regulation of cellular processes (Guillén-Boixet et al., 2020; Molliex et al., 2015; Yang et al., 2020).

In addition, recent studies have shown that most of the proteins whose aggregation is implicated in neurodegeneration undergo phase separation and that aberrant LLPS could be a driver of disease progression (Alberti and Hyman, 2021; Banani et al., 2017; Ray et al., 2020; Shin and Brangwynne, 2017; Wegmann et al., 2018; Wolozin and Ivanov, 2019). Tu's team showed that H_2_O_2_-induced changes in methionine oxidation control LLPS of ataxin-2 (Kato et al., 2019). This study supports the possibility that even cysteine redox changes could control LLPS. RSH to RSSH transformation instead of RSH to RSOH to RSO_x_H transformation could have different effects on proteins that undergo LLPS, regulating their function by changing their basic biophysical properties and, thus, their phase separation.

Finally, RSSH modification could affect other posttranslational modifications. Neighboring S, T, or Y residues could be affected by the bulkiness and negative charge of sulfur and be less prone to phosphorylation. Cross talk between cysteine reactivity and phosphorylation has been recently shown by Cravatt's group (Kemper et al., 2022). In addition, RSSH modification could have effects on signaling *via* other cysteine posttranslational modifications, such as S-nitrosation and S-acylation. All of these processes could have profound effects on cellular signaling, and so, even if persulfidation is just protective, it is still a modification that affects cellular function.

The relatively new field of signaling by protein persulfidation still has many unanswered questions: the mechanism of persulfide formation and transpersulfidation and the identification of “protein persulfidases,” the improvement of methods to monitor RSSH changes and identify protein targets, and understanding the mechanisms through which this modification controls important (patho)physiological functions. Once this knowledge is obtained, it will pave the way for the development of new drugs that could be used to improve health. Therefore, this exciting field of research will remain a hot topic for many years to come.

## Abbreviations Used

α-KB: α-ketobutyrate
α-KG: α-ketoglutarate
3-MP: 3-mercaptopyruvate
AD: Alzheimer's disease
BTA: biotin thiol assay
CAT: cysteine aminotransferase
CBS: cystathionine β-synthase
CSE or CTH: cystathionine γ-lyase
Cys-tRNA: cysteinyl-tRNA
Cyt C: cytochrome c
DTT: dithiothreitol
EGF: epidermal growth factor
EGFR: epidermal growth factor receptor
ETHE1: persulfide dioxygenase
FXN: frataxin
GPX4: glutathione peroxidase 4
GR: glutathione reductase
Grx: glutaredoxin
GSH: glutathione
GSK3β: glycogen synthase kinase 3β
GSSH: glutathione persulfide
H_2_O_2_: hydrogen peroxide
H_2_S: hydrogen sulfide
ISCU: iron–sulfur cluster assembly scaffold protein
L^•^: lipid radicals
LLPS: liquid‒liquid phase separation
LMW: low molecular weight
LO^•^: lkoxyl radical
MAPT: microtubule-associated protein tau
MPST: mercaptopyruvate sulfurtransferase
MS: mass spectrometry
MSBT: methylsulfonyl benzothiazole
MSBT-A: (benzothiazole-2-sulfonyl)-acetic acid
NBD-Cl: 4-chloro-7-nitrobenzofuran
NFS: cysteine desulfurase
NOX: NADPH oxidase
PLP: pyridoxal 5′-phosphate
ProPerDP: protein persulfide detection protocol
Prx: peroxiredoxin
qPerS-SID: quantitative persulfide-site identification
QTRP: quantitative thiol reactivity profiling
RBC: red blood cell
RNS: reactive nitrogen species
ROO^•^: peroxyl radical
ROS: reactive oxygen species
RSH: thiol groups
RS(H): S = thiosulfoxide
RSO_2_H: sulfinic acids
RSO_3_H: sulfonic acids
RSOH: protein sulfenylation
RSSH: protein persulfidation
RSSO_2_H: perthiosulfinic acids
RSSO_3_H: perthiosulfonic acids
SELENBP1 or SBP1: methanethiol oxidase
SO: sulfite oxidase
SQR: sulfide:quinone oxidoreductase
TCEP: tris (2-carboxyethyl) phosphine
ThiI: tRNA sulfur transferase
TRP14: thioredoxin-related protein of 14 kDa
Trx: thioredoxin
TrxR: thioredoxin reductase
TST: thiosulfate sulfur transferase
TSTD1: thiosulfate sulfur transferase-like domain-containing 1 protein
